# Temporal Alterations in Mitochondrial β-Oxidation and Oxidative Stress Aggravate Chronic Kidney Disease Development in 5/6 Nephrectomy Induced Renal Damage

**DOI:** 10.3390/ijms21186512

**Published:** 2020-09-06

**Authors:** Omar Emiliano Aparicio-Trejo, Pedro Rojas-Morales, Sabino Hazael Avila-Rojas, Juan Carlos León-Contreras, Rogelio Hernández-Pando, Alexis Paulina Jiménez-Uribe, Rodrigo Prieto-Carrasco, Laura Gabriela Sánchez-Lozada, José Pedraza-Chaverri, Edilia Tapia

**Affiliations:** 1 Department of Cardio-Renal Pathophysiology, National Institute of Cardiology “Ignacio Chávez”, Mexico City 14080, Mexico; emilianoaparicio91@gmail.com (O.E.A.-T.); pedrorojasm@outlook.com (P.R.-M.); lgsanchezlozada@gmail.com (L.G.S.-L.); 2 Department of Biology, Faculty of Chemistry, National Autonomous University of Mexico (UNAM), Mexico City 04510, Mexico; shaggy_goose@hotmail.com (S.H.A.-R.); athumbix@gmail.com (A.P.J.-U.); rp_2811@hotmail.com (R.P.-C.); pedraza@unam.mx (J.P.-C.); 3 Experimental Pathology Section, National Institute of Medical Sciences and Nutrition “Salvador Zubirán”, Mexico City 14000, Mexico; jcleonc@hotmail.com (J.C.L.-C.); rhdezpando@hotmail.com (R.H.-P.)

**Keywords:** chronic kidney disease, 5/6 nephrectomy, β-oxidation impairment, mitochondrial damage

## Abstract

Five-sixths nephrectomy (5/6Nx) model is widely used for studying the mechanisms involved in chronic kidney disease (CKD) progression, a kidney pathology that has increased dramatically in recent years. Mitochondrial impairment is a key mechanism that aggravates CKD progression; however, the information on mitochondrial bioenergetics and redox alterations along a time course in a 5/6Nx model is still limited and in some cases contradictory. Therefore, we performed for the first time a time-course study of mitochondrial alterations by high-resolution respirometry in the 5/6Nx model. Our results show a decrease in mitochondrial β-oxidation at early times, as well as a permanent impairment in adenosine triphosphate (ATP) production in CI-linked respiration, a permanent oxidative state in mitochondria and decoupling of these organelles. These pathological alterations are linked to the early decrease in complex I and ATP synthase activities and to the further decrease in complex III activity. Therefore, our results may suggest that mitochondrial bioenergetics impairment is an early event in renal damage, whose persistence in time aggravates CKD development in the 5/6Nx model.

## 1. Introduction

Chronic kidney disease (CKD) is characterized by the progressive loss of nephrons, glomerular filtration rate (GFR) reduction, and the increase in renal damage markers during at least 3 months, generating progressive deterioration of the kidney function [1]. Although CKD has increased dramatically in the last few years and its death rate is expected to continue increasing [2,3], the mechanisms of CKD progression over time are only partially known [4] and the current clinical treatments in many cases fail to prevent illness progression [5]. Therefore, there is an urgent need for knowing the molecular mechanisms involved in CKD to prevent or delay the progression of this illness. Renal mass reduction models, like 5/6 nephrectomy (5/6Nx), are widely used for producing a cycle of progressive deterioration in the kidney, which emulates clinical CKD [6,7]. Therefore, 5/6Nx has been used for studying the mechanisms involved in CKD progression [6,7].

On the other hand, renal function highly depends on mitochondrial function, making mitochondrial pathologies a common factor in the development of various kidney diseases [8,9]. Moreover, recent works suggest that renal mitochondrial alterations participate in the progression of several types of CKD in non-diabetic and in diabetic contexts [4,10,11,12,13]. We recently showed that in folic acid-induced renal damage, mitochondria suffer pathological alterations, especially in bioenergetics and fatty acids (FA) β-oxidation parameters, that change along the transition to CKD [10]. Although in the 5/6Nx model it has been shown that renal mass reduction induces mitochondrial bioenergetics alterations in organs like the heart [14] and skeletal muscle [11], our knowledge of renal mitochondrial disorders and their participation in CKD progression is limited because there is no information about the mitochondrial bioenergetics and redox state alterations over a time-course [4], in addition to the fact that the data on bioenergetics measurements and mitochondrial oxygen consumption made by low-resolution oximetry are scarce and in some cases are contradictory [15,16,17]. In this regard, our research group has previously demonstrated that 24 h after 5/6Nx, the early adaptive hemodynamic response induces alterations in mitochondrial bioenergetics, decreasing adenosine triphosphate (ATP) production [18]. The hemodynamic changes persist along the CKD progression [6], suggesting that mitochondrial pathological changes at early times may also persist and be parallel to the changes in renal oxygenation and perfusion, mainly present at early times (in the interval between the first hours and day 28) [7,19,20,21,22,23]. Therefore, the study of mitochondrial alterations in this interval would be of interest to elucidate the mechanisms involved in mitochondrial dysfunction and their role in CKD development in this model.

Consequently, in the present study, we characterize for the first time the time course of mitochondrial bioenergetics alterations in the remnant kidney during days 2 to 28 of nephrectomy. We also characterize for the first time the alterations in FA β-oxidation and their relationship with oxidative stress in mitochondria, and their possible association to the pathological changes in renal hemodynamics parameters associated with CKD development in this model.

## 2. Results

### 2.1. Renal Function Loss Induced by 5/6 Nephrectomy

**Figure 1:**
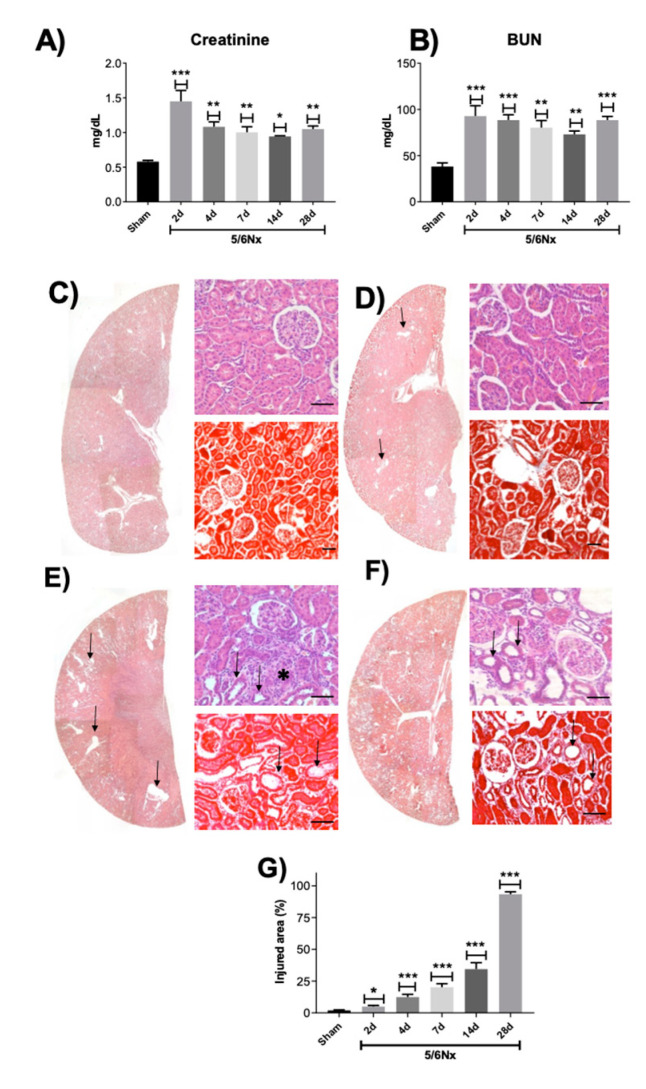
Markers of renal function of the experimental groups. (**A**) Creatinine and (**B**) blood urea nitrogen (BUN) in plasma. (**C**–**F**) Representative micrographs of kidney from different time points, low power (left panel), hematoxylin/eosin (H&E, right upper figure), and Masson trichrome staining (right bottom figure). (**C**) Normal kidney histology from Sham animal. (**D**) On day 7 of 5/6 nephrectomy (5/Nx), there are focal areas of tubular atrophy (black arrows). (**E**) Three weeks after nephrectomy, there were more and bigger areas of tubular atrophy (black arrows) and mild interstitial inflammatory infiltrate (asterisk). (**F**) After four weeks of nephrectomy, extensive areas of the kidney were affected showing tubular epithelium atrophy (black arrows), interstitial inflammation with mild fibrosis. (**G**) The automated morphometry measurement showed progressive areas of kidney injury. Data are mean ± SEM, *n* = 4. * *p* < 0.05, ** *p* < 0.01, *** *p* < 0.001 vs. Sham, Dunnett test. Scale bar = 100 µ.

We previously reported that 5/6Nx induced the increase in the renal damage markers blood urea nitrogen (BUN) and creatinine in plasma 24 h post-surgery [18]. Figure 1A,B shows that both markers remain elevated on day 2 and show a partial recovery in serum creatinine from days 4 to 28 in the 5/6Nx group, confirming the maintenance of renal damage. Furthermore, histology evaluation by hematoxylin and eosin (H&E) and Masson trichrome staining of remnant renal mass shows on day 2 small focal areas of proximal convoluted tubular atrophy manifested by flattened epithelial cells with some necrotic cells. Some of these tubules exhibited hyaline casts in their lumens and were surrounded by interstitial edema with scarce inflammatory cells (Figure 1D). These focal areas increase progressively with time in size and number, as shown by the automated morphometry measurements (Figure 1C–G). On day 28 post-surgery, the kidney damage was extensive, the epithelial tubular atrophy was more accentuated, as well as the interstitial inflammation that showed more lymphocytes and mild fibrosis, as well as thickening of the muscular wall of arterioles that reduced their lumens and glomeruli with increase of mesangial cellularity and mild fibrosis (Figure 1F,G).

**Figure 2:**
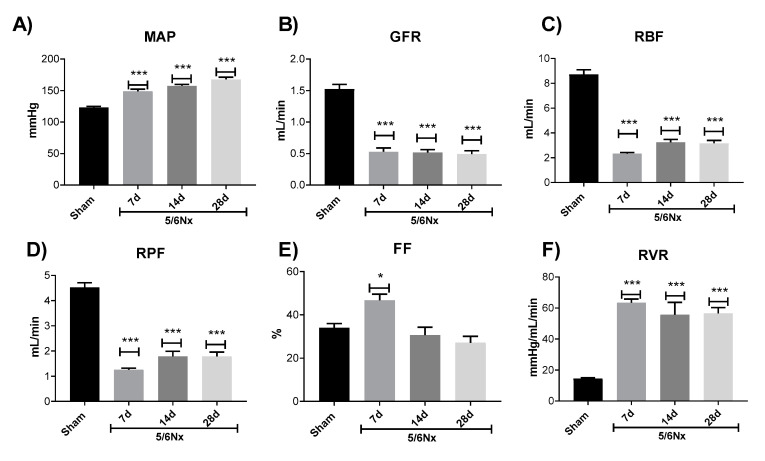
Chronic renal damage induced by 5/6 nephrectomy (5/6Nx). Temporal evaluation of hemodynamic parameters: (**A**) mean arterial pressure (MAP), (**B**) glomerular filtration rate (GFR), (**C**) renal blood flow (RBF), (**D**) renal plasma flow (RPF), (**E**) filtration fraction (FF), (**F**) renal vascular resistance (RVR). Data are mean ± SEM, *n* = 4–8. * *p* < 0.05, *** *p* < 0.001 vs. Sham, Dunnett test. d = days after nephrectomy surgery, Sham = simulated operation-control group.

To confirm CKD development, we evaluated the GFR and the hemodynamics parameters on day 7. Mean arterial pressure (MAP) increases (Figure 2A), whereas GFR (Figure 2B), renal blood flow (RBF, Figure 2C), and renal plasma flow (RPF, Figure 2D) decrease in the 5/6Nx group on day 7 that remains until day 28 (Figure 2). Interestingly, the filtration fraction (FF) slightly increases on day 7 with respect to the Sham group (Figure 2E) probably as a result of renal vasoconstriction also observed at that time point (Figure 2F). However, it is followed by the FF reduction on days 14 to 28 (Figure 2E). Likewise, there is renal vascular resistance (RVR) increase (Figure 2F) on days 7 to 28 in the 5/6Nx group. Together these data confirm that 5/6Nx significantly reduces renal function in the remnant kidney, which ultimately will lead to CKD development in this model [6,17].

### 2.2. 5/6Nx Induces a Permanent Decrease in Oxidative Phosphorylation (OXPHOS) Capacity in CI-Linked Respiration and a Progressive Decoupling in Mitochondrial β-Oxidation

**Figure 3:**
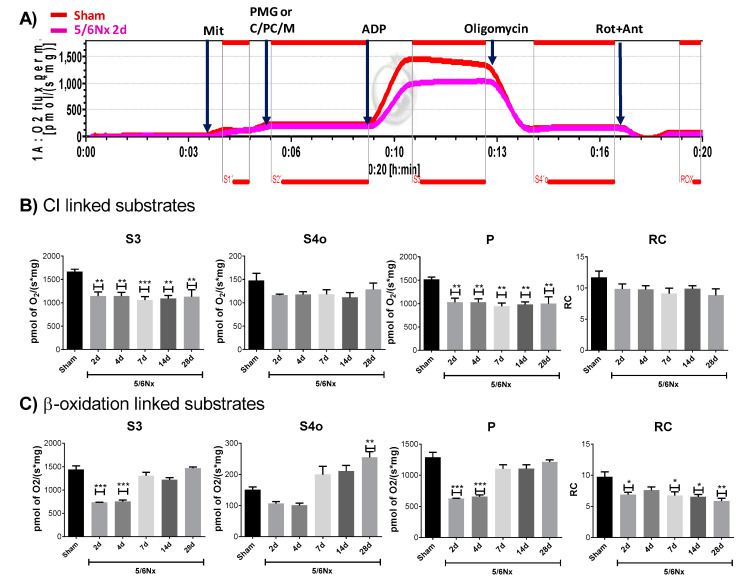
Mitochondrial respiratory parameters in renal remaining mass. (**A**) Schematic representations of the protocol used to determine the respiratory parameters. Red line corresponds to the rate of oxygen (O_2_) consumption of the Sham group; meanwhile, the purple line corresponds to the rate of O_2_ consumption of the nephrectomy group on day 2. Then, 5 mM pyruvate, 2 mM malate, and 10 mM glutamate were used as CI-linked substrates and 2 mM L-carnitine plus 2 μM palmitoyl-L-carnitine plus 2 mM malate (C/PC/M) were used as β-oxidation-linked substrates. Blue arrows represent the additions. ADP = adenosine diphosphate, CI = complex I, L = leak of respiration, Mit = mitochondria, Rot = rotenone, Ant = antimycin A, ROX = residual non-mitochondrial respiration. (**B**) Respiratory parameter of CI-linked respiration: state 3 (S3), state 4 induced by oligomycin (S4o), oxidative phosphorylation (OXPHOS) associated respiration (P), and respiratory control (RC). Data are mean ± SEM, *n* = 4–6. ** *p* < 0.01 vs. Sham, Dunnett test. (**C**) Respiratory parameter of β-oxidation-linked respiration: S3, S4o, P, and RC. Data are mean ± SEM, *n* = 4–6. * *p* < 0.05, ** *p* < 0.01, and *** *p* < 0.001 vs. Sham, Dunnett test. d = days after nephrectomy surgery, Sham = simulated operation-control group, 5/6Nx= 5/6 nephrectomy.

We previously demonstrated, by low-resolution respirometry, that after 24 h of 5/6Nx surgery, the remnant kidney has a reduction in state 3 (S3) respiration and adenosine diphosphate (ADP)/oxygen (ADP/O) ratio, which leads to a reduction in the respiratory control (RC) in complex I (CI)-linked respiration [18]. However, low-resolution respirometry was unable to detect changes in the nephrectomized group at later times [17], despite the fact that numerous studies show that after 4, 8, 12, and 13 weeks, 5/6Nx rats have lower ATP content and lower mitochondrial membrane potential (ΔΨm) in comparison with Sham-operated rats, as well as cristae definition loss and mitochondrial swelling [19,20,23,24], suggesting that mitochondrial respiratory alterations are still present during the progression of the disease. Therefore, we used a high-resolution respirometry protocol to evaluate the respiratory parameters in CI-linked respiration in mitochondria isolated from the remnant kidney (Figure 3A). The 5/6Nx group showed a permanent reduction in S3 and in OXPHOS associated capacity (P) values at all time points evaluated (Figure 3B), implying a reduction in mitochondrial ATP production by CI-linked substrates with respect to Sham. No changes were observed in oligomycin-induced state 4 (S4o) respiration and RC values in CI-linked substrates (Figure 3B). The lack of change in S4o in CI-linked respiration implies that the leak of respiration, mitochondrial processes that consume ΔΨm without contributing to ATP production [25], are not significantly affected in the time interval evaluated, which explain the maintain of mitochondrial coupling in CI-linked respiration (Figure 3B).

On the other hand, mitochondrial ATP production in high energy demanding segments like the proximal tubule (PT) is principally sustained by β-oxidation of FA, like palmitate that represents ~25% of total FAs in plasma [26]. Likewise, proteomic and Western blot analysis of the remnant kidney in 5/6Nx rats shows a reduction in FA β-oxidation–related proteins on day 28 [22] and we recently reported in a model of kidney damage induced by folic acid, that the impairment in OXPHOS capacity and the decrease in β-oxidation favor the transition from AKI to CKD [10]. Therefore, to evaluate if the observed mitochondrial impairment is linked to β-oxidation dysfunction, we temporally evaluated the respiratory parameters by feeding with palmitate and using high resolution respirometry. S3 and P values in β-oxidation–linked respiration are strongly reduced on days 2 and 4 after surgery and have a further increment on day 7 (Figure 3C). However, S4o also progressively increases, which induces the reduction in RC at all evaluated time points (Figure 3C). Collectively, these results show a reduction in mitochondrial ATP production linked to β-oxidation at early time points (days 2 and 4) in the 5/6Nx group and a recovery on day 7 that occurs at the expense of an increase in mitochondrial decoupling, since respiration leaking progressively increases.

**Figure 4:**
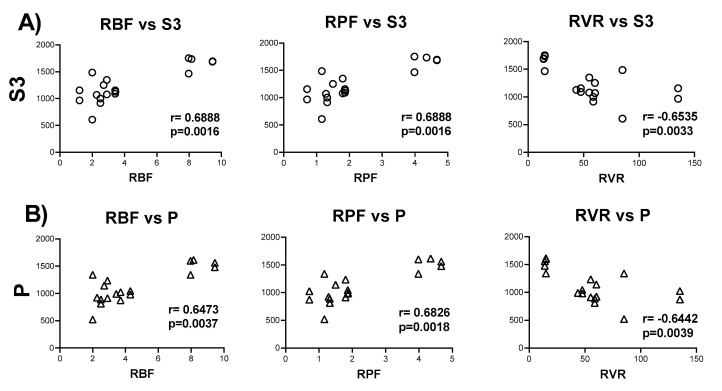
Pearson correlations among changes in energy parameters and hemodynamics parameters. Pearson correlation among the alteration in the nephrectomized group in the respiratory parameters in complex I-linked respiration (**A**) state 3 (S3) and (**B**) oxidative phosphorylation (OXPHOS)-associated respiration (P), and the changes in hemodynamics parameters on days 7, 14, and 28 after surgery. RBF = renal blood flow; RPF = renal plasma flow; RVR = renal vascular resistance.

Finally, to evaluate if there is a correlation among the changes in bioenergetics parameters in CI-linked respiration and hemodynamics alterations, we computed Pearson correlations. Interestingly the decrease in S3 shows positive correlation with the RBF and RPF, as well as negative correlation with RVR (Figure 4A). Likewise, P, whose value indirectly indicates the mitochondrial ATP synthesis, shows a very similar behavior as S3, with positive correlation with RBF and RPF and negative correlation with RVR (Figure 4B).

### 2.3. 5/6Nx-Induced Decrease in OXPHOS Capacity is Related to Mitochondrial Depolarization by CI and CIII Activity Decrease

**Figure 5:**
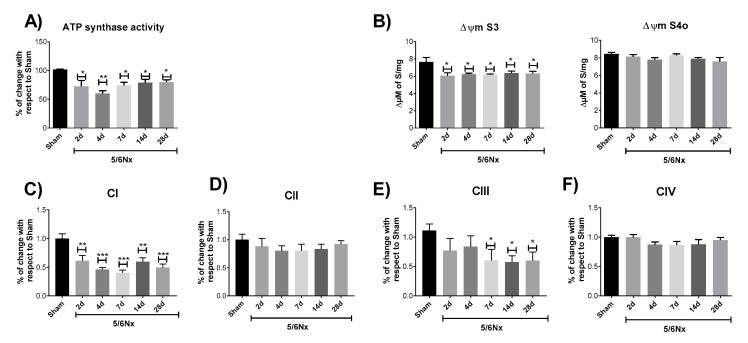
Changes in oxidative phosphorylation (OXPHOS) system elements and membrane potential (Δψm). (**A**) ATP synthase activity. (**B**) Change in Δψm in state 3 (S3) and Δψm in state 4 induced by oligomycin (S4o). (**C**) Complex I (CI); (**D**) complex II (CII); (**E**) complex III (CIII); and (**F**) complex IV (IV). Data are mean ± SEM, *n* = 4–7. * *p* < 0.05, ** *p* < 0.01, and *** *p* < 0.001 vs. Sham, Dunnett test. d = days after nephrectomy surgery, Sham = simulated operation-control group; 5/6Nx= 5/6 nephrectomy.

To determine if the reduction observed in OXPHOS capacity (Figure 3B,C) is related to changes in the activity of OXPHOS components activity, ATP synthase activity was evaluated. The 5/6Nx group shows a permanent reduction in ATP synthase activity (Figure 5A), together with a reduction in Δψm in S3 at all evaluated time points (Figure 5B), which agrees with the reduction in P values (Figure 3B). Furthermore, the 5/6Nx group shows a permanent reduction since day 2 in CI activity (Figure 5C) and a decrease in complex III (CIII) activity on days 7, 14, and 28 after surgery (Figure 5F). However, no changes were found in the evaluated times in complex II (CII, Figure 5D) and complex IV (CIV, Figure 5F) activities with respect to the Sham group. This lack of change in CII and CIV was also reported in the AKI-to-CKD transition model induced by folic acid [10], and has been related to a higher abundance of cysteine residues in CI and CIII, making these complexes more susceptible to oxidative stress [10,27,28,29]. Furthermore, our results are congruent with the reports by Lash et al. [15], who observed a slight increase in S3 in CII-linked respiration on day 10 of nephrectomy. Together, these results imply that in the remnant kidney, the reduction in mitochondrial ATP production is triggered by Δψm depolarization, induced by activity impairment of CI and CIII.

### 2.4. Mitochondrial Decoupling Induced by 5/6Nx is Related to a Pro-oxidative State in Mitochondria

**Figure 6:**
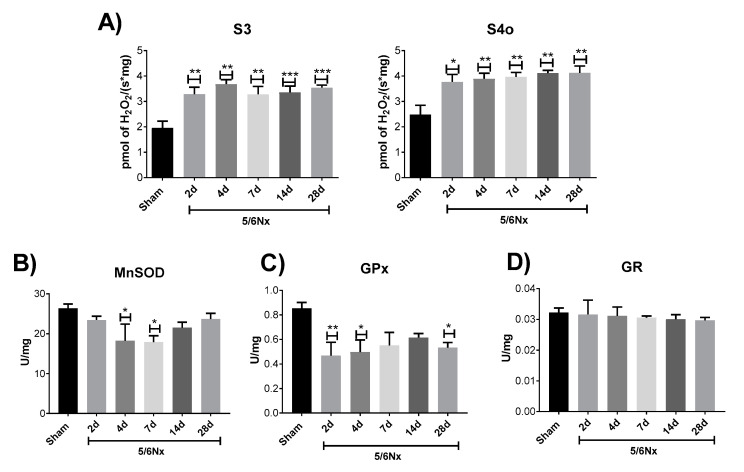
Oxidative stress in mitochondria of the remaining renal mass. (**A**) Hydrogen peroxide (H_2_O_2_) production rates in isolated mitochondria in state 3 (S3) and state 4 induced by oligomycin (S4o). (**B**) Activity of the antioxidant enzymes Mn superoxide dismutase (MnSOD), glutathione peroxidase (GPx), and glutathione reductase (GR) in isolated mitochondria. Data are mean ± SEM, *n* = 5–6. * *p* < 0.05, ** *p* < 0.01, and *** *p* < 0.001 vs. Sham. d = days after nephrectomy surgery, Sham = simulated operation-control group; 5/6Nx= 5/6 nephrectomy.

We previously demonstrated that at 24 h after nephrectomy, the alteration in CI-linked respiration triggers the increase in mitochondrial hydrogen peroxide (H_2_O_2_) production and oxidative stress [18]. Moreover, the decrease in ΔΨm and decoupling often trigger mitochondrial reactive oxygen species (ROS) overproduction [30,31]. Therefore, we evaluate if bioenergetics alterations are linked to increased ROS production by mitochondria. 5/6Nx induces a permanent increase in mitochondrial H_2_O_2_ production in S3 and S4o at all the evaluated time points (Figure 6A). Likewise, the evaluation of antioxidant enzymes in mitochondria shows a reduction in manganese superoxide dismutase (MnSOD) activity on days 4 and 7 (Figure 6B) and in glutathione peroxidase (GPx) activity on days 2, 4, and 28 with respect to the Sham group (Figure 6C). No significant changes were found in glutathione reductase (GR) activity at all evaluated time points (Figure 6D), which could indicate a differential susceptibility of mitochondrial antioxidant enzymes to oxidative stress. Interestingly, this has also been observed in the folic acid induced kidney damage model [29]. However, more in depth studies are still necessary. Together, these data show a persistent increase in mitochondrial oxidative stress in the remnant kidney.

### 2.5. 5/6Nx-Induced Temporal Alterations in Mitochondrial Ultrastructure

**Figure 7:**
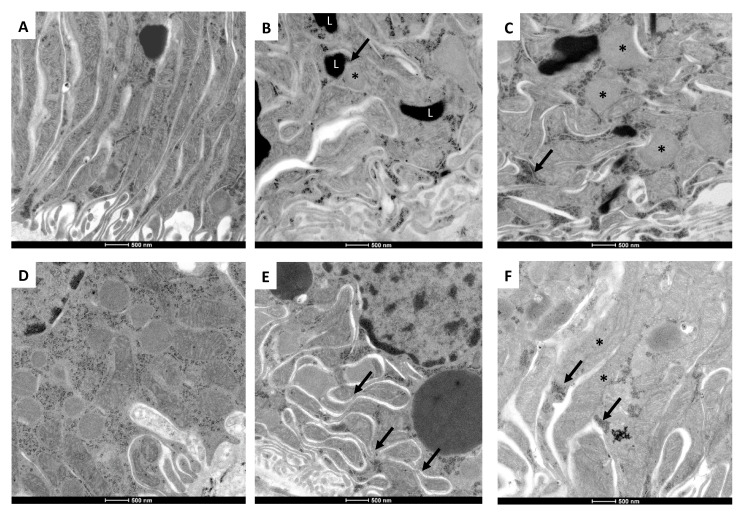
Representative electron microscopy micrographs of mitochondria from tubular epithelial cells at different time points. (**A**) Control Sham animal kidneys show normal mitochondrial structure. (**B**) On day 2 of nephrectomy, mitochondria show cristae effacement and there are numerous lysosomes (L), one small round mitochondria (asterisk) is contacting with a lysosome (mitophagy, arrow). (**C**) More round small mitochondria (asterisks), lysosomes, and numerous free ribosomes (arrow) are seen on day 4 of nephrectomy. (**D**) A similar ultrastructural pattern is observed on day 7 of nephrectomy, some well-preserved mitochondria are surrounded by numerous small round mitochondria with cristae effacement. (**E**) On day 14 of nephrectomy, besides small mitochondria with cristae effacement, many fused mitochondria are also frequent (arrows) and large lysosomes. (**F**) On day 28 of nephrectomy, fused mitochondria are more common, as well as small round mitochondria and cumuli of free ribosomes (arrows).

The ultrastructural morphology of mitochondria is evaluated in the epithelial cells from the proximal convoluted tubules at the different selected time points. In comparison with control animals that show numerous and long well-conformed mitochondria located in the basal cytoplasmic area (Figure 7A), tubular epithelial cells on day 2 of nephrectomy show numerous small round-shaped mitochondria with cristae effacement, alternating with larger irregular-shaped swollen mitochondria with cristae disruption or effacement (Figure 7B). There are numerous lysosomes and some of them are attached to mitochondria (mitophagy). On days 4 (Figure 7C) and 7 (Figure 7D) of nephrectomy, these ultrastructural changes are more accentuated, particularly numerous small mitochondria with cristae effacement, lysosomes, and autophagosomes, as well as many free ribosomes around mitochondria. On day 14 of nephrectomy, there are many small mitochondria with cristae effacement, but fused mitochondria are also common (Figure 7E); while on day 28 of nephrectomy, fused mitochondria are more common, as well as large lysosomes and cumuli of free ribosomes (Figure 7F). Thus, it seems that on days 7 and 14 of nephrectomy, there is substantial mitochondrial damage with active fission activity and mitophagy, while at one month of nephrectomy, there is more mitochondrial fusion, but there are still many small round mitochondria with cristae effacement and some normal-shaped mitochondria with apparently active protein synthesis, supported by the existence of numerous free ribosomes.

## 3. Discussion

In subtotal nephrectomy models, the hemodynamics alterations appear immediately after surgery [32], leading to an increase in single nephron GFR (snGFR), in Na^+^/K^+^ ATPase activity, and in energy expenditure [7]. Nephron loss also triggers a compensatory hypertrophy process in the remnant kidney, increasing the synthesis of biomolecules and growth factors since the beginning of nephrectomy [33,34,35]. Together, hyperfiltration and hypertrophy generate excessive energy demand at early times, especially in the PT, which can induce stress in ATP sources, especially in mitochondria [7,18,22]. Additionally, changes in renal perfusion and oxygenation can induce mitochondrial metabolic changes [7,19,20,21,22,23]. Furthermore, the subsequent over-activation of the renin–angiotensin–aldosterone system (RAAS) can induce further mitochondrial damage, like Δψm depolarization and increase in ROS production, which has been related to nicotinamide adenine dinucleotide phosphate hydrogen (NADPH) oxidase and nuclear factor-*kappa* B (NF-κB) pathways activation [4,36,37]. This is in agreement with our previous report, where we showed that 24 h after 5/6Nx, bioenergetics alterations in mitochondria of the remnant kidney mass lead to a reduction in mitochondrial ATP production [18]. Although in 5/6Nx, the initial adaptations develop to compensate for the renal mass reduction, over time they are maladaptive, producing a cycle of progressive deterioration in the kidney, which results in renal structural damage and progression to CKD [6,7]. In the present study, we confirm the permanence of the renal disease induced by subtotal renal ablation. As we show in Figure 1A,B, the increase in classical renal damage markers BUN and creatinine persists along time in the 5/6Nx group. Moreover, nephron structural damage in the remnant kidney and fibrotic processes progressively increase with time (Figure 1C–G). In fact, on day 7 after surgery, we observed hemodynamic changes characteristic of CKD, like the increase in MAP (Figure 2A), that remains along the time. Interestingly, FF in the 5/6Nx group increased at this time (Figure 2E), which can be explained as part of a compensatory mechanism triggered to compensate renal mass loss. However, as shown by the decrease in GFR, RBF, and RPF (Figure 2B–D), the compensatory increase on day 7 in GFR is not enough to maintain renal function.

There is a consensus that mitochondria fail in maintaining the ATP supply in remnant nephrons [7,38,39]. Reduced perfusion pressure induced by increased vasoconstriction is a well-known mechanism that induces the concomitant reduction in cellular ATP, altering adenine-nucleotides’ metabolism [40]. In the present study, RBF falls 70% on day 7 of 5/6Nx and remains low on days 14 and 28. On the contrary, RVR is increased by approximately 3 times at all evaluated time points (Figure 2). Moreover, we observed a positive correlation among the 2 principal respiratory parameters associated to ATP production, S3 and P, and the changes in RBF (Figure 4A,B). Although the r values are still low (Figure 4), which may be associated with the low number of replicates in the respirometry assays. Collectively, these findings show that the initial injury insult reduces ATP renal concentrations and affects mitochondrial function and structure, as shown in the ultrastructural study that exhibits mitochondrial fission, numerous small round mitochondria with cristae effacement, and lysosomes contacting mitochondria denoting mitophagy, which is observed in all the studied time points with some mitochondrial fusion after two weeks of kidney resection (Figure 7). Over time, it is possible that the impairment in mitochondrial ATP production might directly affect renal blood supply. In addition, it has been shown that renal ischemic injury permanently damages renal microvasculature perpetuating tissue ischemia [41], likely contributing to further damage to the mitochondrial ATP production.

As we mentioned, currently, literature agrees in the fact that mitochondrial adaptations after nephrectomy are not enough to maintain the ATP supply [7,38,39]. However, the mechanism and progression of mitochondrial alterations, especially in the time interval between day 1 and day 28, is still unclear [4]. The lack of change in respiratory parameters previously reported during this interval [17] can be explained by the resolution limitation of classical Oxygraph protocols used in those works, where OXPHOS capacity (P) and State 3 are frequently underestimated and S4 is overestimated, avoiding the discernment between subtle changes [25]. Therefore, we use high-resolution respirometry to evaluate mitochondrial bioenergetics alterations. Our results show that the fall in mitochondrial ATP production in CI-linked respiration is due to a reduction in P and in S3 values (Figure 3B) at all evaluated time points, implying that early mitochondrial damage reported at 24 h [18] is preserved along the CKD transition. This is in agreement with the reduction in ATP concentrations and in the concentrations of the β subunit of ATP synthase in kidney cortex of rats observed on day 28 of 5/6Nx [23]. Our results show that mitochondrial OXPHOS capacity reduction (Figure 3B) is the result of a decrease in Δψm in the S3 (Figure 5B) respiratory state, in which oxygen consumption is predominantly used for ATP production. Thus, the lower Δψm (Figure 5B) results in lower ATP synthase activity (Figure 5A). This reduction in Δψm can be attributable to CI activity reduction observed since day 2 (Figure 5C), but also to the posterior decrease in CIII activity observed since day 7 (Figure 5E), implying that CI impairment precedes CIII impairment. Interestingly, this phenomenon was also observed in the AKI-to-CKD transition model induced by folic acid administration [10] and can be associated with a higher susceptibility of CI to suffer post-transcriptional modifications induced by oxidative stress [27,28,29]. Moreover, these data agree with the decline in CI and CIII activities, and lower ATP synthase β subunit and NDUFB8 CI subunit levels detected on day 56 in 5/6Nx rats [24]. In fact, several groups reported in 5/6Nx groups that after day 28, the reduction in mitochondrial biogenesis triggers CI activity decrease [19,20,22,24,42]. However, at early times, namely on days 1, 10, and 14 of nephrectomy, studies did not find increase in mtDNA, in mtRNA, in mitochondrial complexes’ subunit levels, or in levels of transporters [19,20,22,24,42], which may suggest that the observed complexes’ activities reduction (Figure 5C,E) is not linked to mitochondrial biogenesis reduction at the early stage.

On the other hand, in this work, we report for the first time in 5/6Nx, a reduction in respiratory parameters S3 and P, and in FA β-oxidation on days 2 and 4 (Figure 3C), showing that ATP production by β-oxidation is also decreased at early time points in this model. This is particularly important because recent studies suggest that impairment in mitochondrial β-oxidation proteins appears to be a common pathology in CKD [5,22,43]. In fact, in folic acid-induced renal damage, the impairment in mitochondrial FA β-oxidation is an early mechanism that promotes CKD transition and the increase in fibrotic processes [10,44]. In concordance, our results show that β-oxidation impairment (Figure 3C) precedes collagen accumulation in the remnant kidney (Figure 1D–F). Additionally, β-oxidation–linked substrates show progressive decrease in mitochondrial coupling (Figure 3C), which partially explains the increase in mitochondrial H_2_O_2_ production observed along the evaluated time interval (Figure 6A). This, together with the reduction in antioxidants enzyme levels in mitochondria (Figure 6B,C), implies a permanent pro-oxidative state in these organelles in the remnant kidney. In fact, on day 10 of 5/6Nx, the decrease in aconitase activity (a mitochondrial oxidative stress indicator) and the increased levels of 4-hydroxy-2-nonenal, have been previously reported [45]. Mitochondria are also more susceptible to be damaged by external oxidants [15], confirming a redox mitochondrial imbalance at early time points after renal ablation. Additionally, recent reports show that a chronic mitochondrial pro-oxidant state persists long-term in 5/6Nx rats, as suggested by elevated MDA levels, decreased GSH levels, and reduced MnSOD activity and protein levels on days 56, 84, and 91 of nephrectomy [19,20,23,24,46]. Furthermore, mitochondrial oxidative stress at a chronic stage strongly induces the inflammatory and fibrotic processes that lead to CKD in this model [18,20,47]. An additional deleterious consequence of a reduction in FA β-oxidation is the accumulation of intrarenal lipids causing lipotoxicity, a mechanism that is detrimental for kidney function [48].

**Figure 8:**
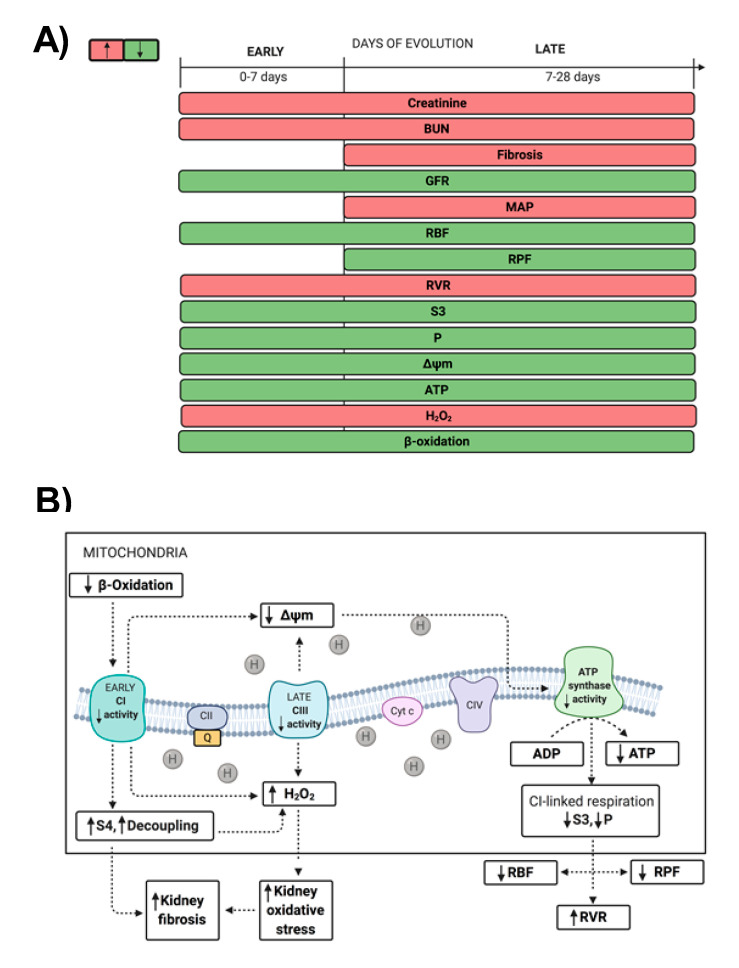
(**A**) Temporal evolution of the pathological change observed in remnant kidney after 5/6 nephrectomy (5/6Nx). To facilitate analysis, two stages can be identified, an early stage (since the first hours and until day 7) and a late stage (days 7 to 28). The red bars represent an increase in the values of the respective parameters, while the green bars represent a decrease. (**B**) Integrative scheme of the mitochondrial pathological changes and their contribution to CKD development in the 5/6Nx model. Up solid line arrow (↑) indicate an increase in the corresponding parameter. Down solid line arrows (↓) indicate a decrease in the corresponding parameter. Dotted arrow indicate the proposed relationships between observed changes. BUN = blood urea nitrogen; CI = mitochondrial complex I; CII = mitochondrial complex II; CIII = mitochondrial complex III; CIV = mitochondrial complex IV; Cyt *c* = cytochrome *c*; GFR = glomerular filtration rate; H_2_O_2_ = mitochondrial hydrogen peroxide; Q = quinone; P = oxidative phosphorylation-associated respiration; S3 = respiratory state 3; S4o = respiratory state 4 induced by oligomycin; RBF = renal blood flow; RPF = renal plasma flow; RVR = renal vascular resistance; MAP = mean arterial pressure; ΔΨm = mitochondrial membrane potential.

As we present in Figure 8A, the pathological changes observed in the remnant kidney can be divided into two time periods for analysis: (1) those that are observable since early times, like hemodynamics alterations that appear immediately after the renal mass loss, including the increase in RVR and glomerular capillaries pressure, as well as the decrease in RBF. These alterations may be tightly related to mitochondrial damage, especially to impairment in OXPHOS capacity and β-oxidation, which persist and are progressive along the evolution of the illness and consist in focal areas of tubular atrophy and interstitial inflammation. Meanwhile, (2) the second appear at later times and are related to the development of CKD, like the increase in MAP and augmented interstitial inflammation with fibrosis, thickening of the muscular layer of arterioles and increase in mesangial widening. As we present in an integrative scheme of mitochondrial alterations (Figure 8B), since early times, 5/6Nx induces the decrease in CI activity and mitochondrial β-oxidation. These changes and the posterior decrease in CIII activity trigger a permanent reduction in Δψm, favoring the reduction in ATP synthase activity and therefore in ATP production. As it can be observed by the reduction in S3 and P values in CI-linked respiration, which correlate with the decrease in RBF and RPF and with the increase in RVR, hemodynamics changes that are closely related to the CKD development in this model. On the other hand, the impairments in complexes’ activities and in β-oxidation also favor the increase in mitochondrial decoupling and in S4o, enhancing the leak of respiration and mitochondrial H_2_O_2_ production. Thus, our results may suggest that the renal increase in ROS and oxidative stress, together with the impairment in β-oxidation can contribute to development of kidney fibrosis and CKD progression in this model.

Although the dramatic reduction in nephrons in a short time produced by renal ablation models, like 5/6Nx, is infrequent in patients, this kind of model is most widely used to characterized CKD progression, because it mimics many pathological processes observed in the human counterpart [7,49]. Additionally, many mechanisms involved in CKD progression in this model are also found in patients, in both diabetic and non-diabetic contexts [6,7]. Although these facts make 5/6Nx a powerful tool to study CKD progression, this model also possesses limitations; for example, genetic polymorphisms in animals and their gender could impact on susceptibility to 5/6Nx. In fact, 129/Sv mice, Wistar rats, and female rats are more susceptible to renal damage than C57BL5 mice, Sprague-Dawley rats, and male rats [50]. Furthermore, the pathological insults involved in the genesis of renal damage can affect the mechanism involved in CKD evolution, as in the case of diabetes, autoimmune diseases, tumors, to name a few [7,49,51]. Therefore, the results we present in this work should be taken with caution if they are to be extrapolated to other models of kidney damage. However, growing evidence suggests that renal mitochondrial alterations participate in the progression of several types of CKD in both non-diabetic and diabetic contexts [4,7,11,12,13,45,52].

The present work may also suggest that mitochondrial focus therapy and especially the use of mitochondria-targeted compounds can prevent or delay the progression of CKD. In fact, as we show in Figure 8, our results show that both CI-CIII dysfunction and β-oxidation impairment would be the principal therapeutic molecular target in mitochondria, since their functional preservation would prevent subsequent mitochondrial alterations in the 5/6Nx model. Although the 5/6Nx model does not fully cover the broad spectrum of pathologies included in CKD, other CKD progression models, like that induced by folic acid [10], also show a reduction in OXPHOS capacity related to CI-CIII and β-oxidation impairment. Furthermore, in this model, the preservation at early times of complexes’ activities by N-acetylcysteine prevents β-oxidation impairment and the subsequent AKI-to-CKD transition [10]. Although the correlation of the information obtained in the experimental models with clinical data of patients in CKD is still poor [53], β-oxidation was recently reported deregulated in patients [44,54] and its downregulation was related with CKD progression by fibrotic processes [44]. Together, these works and our results may suggest that mitochondrial complexes and β-oxidation target therapy can potentially prevent kidney damage progression in multiple kinds of CKD. In fact, in the 5/6Nx model, mitochondrial targets therapies like administration of Mito-TEMPO [55], carnitine [56], and resveratrol [23], antioxidants able to induce mitochondrial biogenesis, can prevent CKD progression. However, deeper studies and improved clinical protocols are still necessary to confirm this hypothesis, with special interest in molecules focused on preventing the loss of the mitochondrial complex’s activities at early stages of kidney damage.

## 4. Materials and Methods

### 4.1. Reagents

Amplex Red, antimycin A, ADP sodium salt, L-arginine, bovine serum albumin (BSA), FA free BSA, carbonyl cyanide m-chlorophenylhydrazone (CCCP), cytochrome *c* from equine heart, D-(+)-glucose, D-mannitol, decylubiquinone (DUB), 2,6-dichlorophenolindophenol sodium salt hydrate (DCPIP), 5,5′-dithio-bis-(2-nitrobenzoic acid) (DTNB), ethylene glycol-bis(2-aminoethylether)-N,N,N′,N′-tetraacetic acid (EGTA), glutathione (GSH), glucose-6-phosphate dehydrogenase, oligomycin, GR, hexokinase, 4-(2-hydroxyethyl)-1-piperazineethanesulfonic acid (HEPES), horseradish peroxidase (HRP), K-lactobionate, L-carnitine, palmitoyl-L-carnitine, manganese (II) chloride (MgCl_2_) tetrahydrate, NADPH, nicotinamide adenine dinucleotide phosphate (NADP^+^), nicotinamide adenine dinucleotide hydrogen (NADH), nitroblue tetrazolium (NBT), paraformaldehyde, potassium cyanide (KCN), rotenone, safranin O, hematoxylin, eosin, sodium succinate dibasic, sodium glutamate, sodium L-ascorbate, sodium malate, sodium pyruvate, sodium dodecyl sulfate (SDS), sucrose, taurine cacodylate buffer, osmium tetraoxide, uranyl acetate, lead citrate, and tetramethyl-p-phenylenediamine (TMPD) were purchased from Sigma-Aldrich (St. Louis, MO, USA). Calcium chloride (CaCl_2_), sodium bicarbonate (NaHCO_3_), ethylenediaminetetraacetic acid disodium salt dihydrate (EDTA), sodium phosphate dibasic (Na_2_HPO_4_), and sodium phosphate monobasic (NaH_2_PO_4_) were purchased from JT Baker (Xalostoc, Edo. Mex., Mexico). Polyfructosan (Inutest^®^) was purchased from Fresenius-Kabi (Linz, Austria). Trichloroacetic acid, sulfuric acid, and anthrone were purchased from Merck (Naucalpan, Edo. Mex., Mexico). Sodium pentobarbital (Sedalphorte^®^, Mexico City, Mexico,), used as sedative, was from Salud y Bienestar Animal S.A. de C.V. (Mexico City, Mexico). Reagents to measure plasma creatinine and BUN were purchased from Spinreact (Girona, Spain).

### 4.2. Experimental Design

The experimental protocol was approved by the Institutional Animal Care and Use Committee (CICUAL) of the National Institute of Cardiology “Ignacio Chávez” (INC/CICUAL/014/2018; approbation date: 11/22/2018) and was conducted according to the Mexican Official Guideline for the use and care of laboratory animals (NOM-062-ZOO-1999) and for the disposal of biological residues (NOM-087-SEMARNAT-SSA1-2002) and The Guide for the Care and Use of Laboratory Animals 8th Edition. Two groups of male Wistar rats with an initial body weight between 250 to 300 g were studied: (1) Sham-operated rats and (2) 5/6Nx group. Five-sixths nephrectomy was performed using the protocol described previously [18]. Rats were housed in a temperature-controlled environment with a 12–12 h light-dark cycle and maintained with water and food ad libitum. The analysis was carried out on days 2, 4, 7, 14, and 28 of 5/6Nx, with *n* = 6/group for each time point. Animals were anesthetized with sodium pentobarbital (60 mg/kg), blood was obtained from abdominal aorta, plasma was separated and stored at 4 °C to determine BUN and plasma creatinine as markers of renal function. Additional groups of Sham and 5/6Nx rats were allocated to perform renal hemodynamic studies on days 7, 14, and 28 (*n* = 8/group).

### 4.3. Biochemical Markers of Renal Damage and Hemodynamic Parameters

The biochemical renal damage markers creatinine and BUN concentrations in plasma were assessed by commercial kits according to manufacturer’s instructions. The evaluation of GFR and hemodynamics parameters was carried out as previously described [18]. Briefly, before each time point follow-up, the animals were anesthetized with sodium pentobarbital (50 mg/kg) and the femoral arteries, jugular veins, trachea, and bladder were catheterized. The body temperature was maintained at 37 °C by means of a thermoregulated table. A femoral artery catheter was used to monitor MAP with a physiological pressure transducer MLT844 (ADInstruments, Colorado Springs, CO, USA), whereas the other femoral catheter was used for blood sampling. The blood samples were taken in microcapillaries and centrifuged in a hematocrit centrifuge (MICRO-MB, Thermo IEC, Thermo Fisher Scientific, Inc., Waltham, MA, USA) and the hematocrit was read with a microcapillary reader (Damon IEC Division Needham Heights, MA, USA). To compensate for bodily fluid loss, 6% BSA in sterile saline solution was infused through the vein and the bladder was catheterized to allow urine sampling. For the evaluation of GFR, rats received an infusion with 5% polyfructosan (in 0.9% saline solution) at a rate of 2.2 mL/h during a 60-min period for equilibrium reach. Then, a sample of blood was taken, and the 30 min urine collection was started. In blood and urine samples, the concentration of polyfructosan was measured at 450 nm by the anthrone method [17] to calculate GFR. Later, the left kidney was exposed by a left subcostal flank incision, immobilized in a Lucite holder, sealed around with 4% agar, and the kidney surface was covered with 0.9% saline solution. RBF was also determined using a transit-time ultrasound flow probe TS420 (Transonic System, Ithaca, NY, USA) placed around the left renal artery. In addition, RVR was calculated according to the formula RVR = MAP/RBF. RPF was calculated with the formula RPF = RBF*(1-hematocrit), whereas the FF was defined as FF = GFR/RPF.

### 4.4. Histology Evaluation and Electron Microscopy

Immediately after animal sacrifice, thin tissue slices of remnant renal mass were fixed by immersion in a mixed solution of 4% paraformaldehyde and 1.5% glutaraldehyde pH = 7.2. A thin tissue slide (1 mm width) was dehydrated and embedded in paraffin, sectioned at 5 μm and stained with H&E and Masson trichrome for each time. To measure kidney damage, each slide was photographed using a camera system that produced an image of the full kidney area, corresponding to an area of 100%. Then, the focal areas of tubular damage and interstitial fibrosis were delimited and measured with the software analyzer (Qwin, Leica, Wetzlar, Germany). Finally, the percentage of the kidney injury surface area was determined. For the ultrastructural electron microscopy evaluation, small tissue fragments of the remnant renal mass were fixed with 2.5% glutaraldehyde in 0.15 M cacodylate buffer, post-fixed with 1% osmium tetroxide, dehydrated with ethyl alcohol in ascending concentrations, and infiltrated in epoxy resin. Ultrathin sections were contrasted with uranyl acetate and lead citrate, and subsequently observed with an electron microscope (Tecnai Spirit BioTwin, FEI, Hillsboro, OR, USA)**.**

### 4.5. Renal Mitochondria Isolation

Renal mitochondria were isolated as previously described [57]. Briefly, mitochondria were isolated by differential centrifugation in 4 °C isolation buffer (225 mM D-mannitol, 75 mM sucrose, 1 mM EDTA, 5 mM HEPES, 0.1% FA free BSA, pH 7.4) The final mitochondrial pellet was resuspended in 180 μL of BSA-free isolation buffer and the mitochondrial total protein was measured by the Lowry method.

### 4.6. Mitochondrial O_2_ Consumption and ΔΨm

Evaluation of mitochondrial O_2_ consumption was performed with a high resolution respirometer (Oxygraph O2k, OROBOROS, Innsbruck, Austria) at 37 °C. Isolated mitochondria (200 µg of total protein) were loaded into the chamber containing 2 mL of respiration buffer MiR05 (0.5 mM EGTA, 3 mM MgCl_2_, 60 mM K-lactobionate, 20 mM taurine, 10 mM KH_2_PO_4_, 20 mM HEPES, 110 mM sucrose, and 1 g/L essentially FA free BSA, pH = 7.4). Electron transport was started by addition of CI-linked substrates (5 mM pyruvate, 2 mM malate, and 10 mM glutamate), CII-linked substrates (10 mM succinate plus 0.5 µM rotenone), or β-oxidation–linked substrates (2 mM L-carnitine, 2 μM palmitoyl-L-carnitine plus 2 mM malate) [10]. Respiration in S3 was achieved by addition of 2.5 mM ADP and S4o by addition of 2.5 μM oligomycin. All parameters were corrected by residual respiration (ROX) values obtained by addition of 0.5 µM rotenone 0.5 µM plus 2.5 µM antimycin A. The RC was defined as the S3/S4o ratio and OXPHOS-associated respiration (P) was defined as S3-S4o [29,58]. All values were normalized by total protein content.

The changes in ΔΨm for the different respiratory states were measured as previously reported [29]. Briefly, the changes in safranin O fluorescence (2 µM) were used to determine the ΔΨm. To stimulate CI, CII, or β-oxidation–linked respiration, the respective substrates were added. ΔΨm in S3 was obtained by addition of 2.5 mM ADP and in S4o by 2.5 μM oligomycin, 5 μM CCCP was added to completely dissipate the ΔΨm and to correct by the non-specific interactions. Results were expressed as the changes in the measurable concentration of safranin O (ΔμM of S) in S3 or S4o with respect to CCCP decoupling, and the results were normalized per milligram of protein (ΔμM of S/mg of protein).

### 4.7. ATP Synthase and Mitochondrial Respiratory Complexes’ Activities

Mitochondrial complexes’ activities were assessed as previously described [29]. Briefly, CI oxidizes NADH while reducing DUB to DUbH_2_, which is then oxidized by DCPIP. Therefore, the decrease in the absorbance of DCPIP at 600 nm is proportional to the activity of CI. CII activity was measured in presence of 2.5 µM rotenone, using the CII’s capacity of reducing DUB. Therefore, the decrease in the absorbance at 600 nm is proportional to the activity of CII. The activity of CIII was evaluated using the increase in absorbance at 550 nm (cytochrome *c* reduction) by addition of DUbH_2_. The activity of CIV was evaluated by addition of 0.5 mM TMPD plus 2 mM ascorbate to respiration medium MiR05 supplemented with 0.5 µM rotenone plus 2.5 µM antimycin A. Oxygen consumption rate was proportional to CIV activity. ATP synthase activity was measured following the reduction of NADP^+^ at 340 nm using an enzyme-linked assay [18]. Absorbance measurements were performed at 37 °C using a Synergy-Biotek microplate reader (Biotek Instruments, Winooski, VT, USA) and the oxygen consumption rate measurements were performed using a high-resolution respirometer (Oxygraph O2k). The specific activity of each complex was determined by the subtraction of the activity in the presence of the appropriate inhibitor from the non-inhibited one. The results are expressed as nmol/min/mg protein.

### 4.8. Mitochondrial H_2_O_2_ Production

Mitochondrial H_2_O_2_ was measured as previously described [29] in an O2k-Fluorometer (OROBOROS, Innsbruck, Austria) using Amplex red as a probe. Isolated mitochondria were resuspended in 2.0 mL of MiR05 plus HRP 0.5 U/mL. Calibration curves were employed to ensure the linearity of the assay and sequential additions were employed to determine the production rate for each state.

### 4.9. Activity of Antioxidant Enzymes in Mitochondria

Isolated mitochondria were used for the measurement of antioxidant enzymes activities as previously described [29]. Briefly, mitochondrial MnSOD activity was evaluated by spectrophotometry at 560 nm using NBT as a probe. GPx activity was measured by the disappearance of NADPH at 340 nm in a coupled reaction with GR. GR activity was evaluated by measuring the disappearance of NADPH at 340 nm.

### 4.10. Statistical Analysis

Data are presented as mean ± standard error of the mean (SEM). They were analyzed by one-way analysis of variance with a subsequent Dunnett test and Pearson’s correlation coefficient (r) was used for testing variables of interest. Both tests were performed using the Graph-Pad Prism 7 software (San Diego, CA, USA). The level of significance was set at *p* < 0.05.

## 5. Conclusions

The subtotal renal ablation triggers early hemodynamics changes that lead to a pathologic state in the mitochondrial CI-linked respiration in the remnant kidney. This pathologic state is characterized by a reduction in OXPHOS capacity, by a decrease in CI activity at the early stage; and at the later stage, by a decrease in in CIII activity. These effects, together with mitochondrial β-oxidation alterations (mitochondrial decoupling), persist over time. Together, our results may suggest that mitochondrial impairment promotes CKD development in this model.
